# Different scientific approaches are needed to generate stronger evidence for population health improvement

**DOI:** 10.1371/journal.pmed.1002639

**Published:** 2018-08-28

**Authors:** Martin White, Jean Adams

**Affiliations:** MRC Epidemiology Unit and Centre for Diet and Activity Research, Institute of Metabolic Sciences, School of Clinical Medicine, University of Cambridge, Cambridge, United Kingdom

## Abstract

In a Perspective, Martin White and Jean Adams discuss challenges in the evaluation of interventions intended to benefit population health.

## The challenge of evaluating low-agency population interventions

Over the last 30 years we have witnessed a growing interest in population approaches to prevention of non-communicable diseases (NCDs) among both policymakers and researchers. The population approach to prevention, where interventions are delivered to all, irrespective of baseline risk, is generally considered more effective than the ‘high-risk’ approach, where interventions are targeted at those at high baseline risk of diseases [1,2]. Furthermore, low-agency population interventions, which place low demands on recipients’ personal resources, are likely to be the most effective and equitable approach to prevention [2]. Examples of low-agency population interventions include taxes on unhealthy commodities (e.g., tobacco, alcohol, gambling, and some foods), regulation of the marketing of these commodities, and structural interventions to make healthier choices easier (e.g., cycle lanes and mandatory installation of seat belts in cars).

The evaluation of low-agency population approaches to prevention presents particular challenges. The nature of these interventions means that they are generally designed and implemented by large organisations such as governments, rather than by researchers. This means that issues of evaluation are often not paramount. By definition, population interventions are delivered to whole populations, meaning that a concurrent control group may not always be obvious. For example, when new national restrictions on food marketing to children are introduced, the only possible concurrent control group is another country—which may differ substantially from the intervention country in ways that undermine its utility as a control group. The lack of an appropriate control also precludes randomisation, even at the group level. In these cases, observational designs making best use of routinely available data and treating the intervention as a ‘natural experiment’ are often the best available evaluative approaches [3].

## The challenge of multiple possibilities

Two examples of evaluations of low-agency population interventions recently published in *PLOS Medicine*, both focussing on the same fiscal policy, illustrate some of the challenges of this approach. Ryota Nakamura [4] and Juan Carlos Caro [5] and their respective colleagues both evaluated a revision to the taxation of sugar-sweetened beverages (SSBs) in Chile, introduced in October 2014, using retrospective, natural experimental designs. The tax reform resulted in an increase in the taxation of SSBs containing at least 6.25 mg/ml of sugar (from 13% to 18%) and a reduction in taxation of SSBs containing less than 6.25 mg/ml of sugar (from 13% to 10%). Both studies used a similar approach to evaluation; both approached the evaluation from an economic perspective, underpinned by theory relating to price elasticities of demand. Both used the same main data source (from a consumer panel representing a stratified random sample of households in urban areas of Chile). But each made different choices in how the data were analysed. Key similarities and differences between the two studies are summarised in Table 1. The two studies came to similar conclusions concerning the overall effectiveness of the policy (weak) and its distributional impacts (more effective in higher socio-economic groups), but the details of their findings differed widely in magnitude and, in some cases, direction. For example, Nakamura et al. found an overall 5.8% decrease in volume (millilitres) of SSBs purchased following introduction of the tax change, whereas Caro et al. found a 1.9% decrease. The decrease was 21.6% for high tax/sugar drinks in Nakamura et al., but only 3.4% in Caro et al.

**Table 1:** Key differences in study design, methods, and main findings between Nakamura et al. [4] and Caro et al. [5].

Methodological choice	Nakamura et al.	Caro et al.
Study design and analytical approach	Time series analysis using fixed effects regression analyses	Pre–post design using random effects regression analyses
Sensitivity analyses	Difference-in-difference regression method Sensitivity of models to different functional forms of time trends	Alternative model specifications (taking account of autocorrelation and two-step models)
Outcome measures	Changes in volume (ml) of household purchases of SSBs Changes in price (pesos/ml) of purchased SSBs by SKU	Changes in volume (ml) of household purchases of SSBs Changes in calories (kcal) of household purchases of SSBs Changes in price (pesos/ml) of purchased SSBs by SKU
Contextual (confounding) factors taken into account	Average monthly temperature Macroeconomic measure: unemployment rates	Seasonality (quarterly indicator variables, not specified) Macroeconomic measures: regional unemployment, population size, supermarket sales, economic index, and construction permits granted
Secondary analyses	Outcomes by SEG (low, middle, high) Outcomes in relation to the announcement of the SSB tax Analysis of the influence of the SSB tax on shopping behaviours—frequency of purchases, use of price promotions	Outcomes by SEG (low, high) Outcomes in relation to the announcement of the SSB tax (data not presented in paper, findings non-significant)
Data sources	Kantar Worldpanel Chile—household shopping panel Nutritional data on sugar content of products from several sources (covering 90% of top-selling SSBs represented in the Kantar dataset), including a large, nationally representative survey, manufacturers’ documents and webpages, and national health authorities’ surveillance systems; nutrition facts panel data from 90% of products purchased	Kantar Worldpanel Chile—household shopping panel Nutritional data on sugar content of products from nutrition facts panel data (79.8% of products), Mintel Latin America (19.9%), or Mintel North America (0.2%), or imputed using a systematic match based on sister products using package description, brand, and manufacturer (<0.1% of each beverage category)
Sample size	2,836 households	2,000 households with 2 months of data, 1,795 with 36 months of data
Time periods for analysis	46 months pre- and 14 months post-implementation of the SSB tax	22 months pre- and 14 months post-implementation of the SSB tax
Main findings	Overall −5.8% change in volume (ml) of SSBs purchased (−21.6% for high tax/sugar drinks, +3% for low tax/sugar drinks, −10% for no tax/sugar drinks) Overall −1.0% change in price (pesos) of SSBs purchased (−0.8% for high tax/sugar drinks, −1.7% for low tax/sugar drinks, +1.7% for no tax/sugar drinks) Differential effects by SEG: bigger effects on volume of high sugar drinks purchased in middle and high SEGs	Overall −1.9% change in volume (ml) of SSBs purchased (−3.4% for high tax/sugar drinks, +10.7% for low tax/sugar drinks, −3.1% for no tax/sugar drinks) Overall −2.3% change in kcal/capita/day purchased (−4.0% for high tax/sugar drinks, −5.3% for no tax/sugar drinks) No overall estimate of price (pesos) change (+3.9% for high tax/sugar ready-to-drink SSBs, +1.5% for low tax/sugar drinks, +1.8% for no tax/sugar drinks) Differential effects by SEG: bigger effects on volume of high sugar drinks purchased in high SEG

SEG, socio-economic group; SKU, stock keeping unit; SSB, sugar-sweetened beverage.

It is not obviously the case that any of the design choices made by either Nakamura et al. [4] or Caro et al. [5] were wrong. They are just different. This illustrates well the challenge of synthesising findings from across evaluations of low-agency population interventions. Alongside researchers’ justifiably choosing a variety of different evaluative designs and analytical strategies, interventions that appear similar may also vary in detail. For example, a wide range of different approaches to SSB taxation have now been implemented internationally [6,7]. In addition, contextual factors are likely to lead to similar interventions achieving different effects in different settings. In these situations, meta-analysis is likely to be challenging; only outcomes common to studies can be analysed and important factors that may influence outcomes will be overlooked. Other, more conceptual, approaches to synthesis may be more appropriate [8].

Disconnected efforts to generate evidence on public health challenges can also be inefficient. In the case of the Chilean SSB tax evaluations, two teams with funding from two different countries led studies in parallel. With greater coordination (and, without doubt, no small measure of imagination and political will), the funding could have been pooled, enabling a wider range of questions to be answered instead of the same set twice. Whilst pragmatic evidence on the effect of SSB taxes on price and sales is now beginning to accumulate [9–14], fewer attempts appear to have been made to investigate wider questions on, for example, reformulation, total diet, health outcomes over different time frames, and the political processes that facilitate and hinder SSB taxes being implemented. Greater coordination of efforts internationally could facilitate more efficient and more strategic approaches to accumulating evidence on SSB taxes, as well as a range of other low-agency population health interventions for NCD prevention.

## The challenge of complexity

Both of the evaluations described above took a relatively simple approach to evaluating a somewhat complex problem. Neither paper presented a theory of change [15] for the intervention they were evaluating. However, it is clear that in both cases the implicit theory of how the intervention was thought to achieve its likely impacts was relatively simplistic: a linear pathway from price change to changes in consumption [16]. Yet both sets of authors also hinted at more complicated mechanisms to explain unexpected findings, such as the socio-economic patterning of outcomes. For example, they suggested that other purchasing behaviours (e.g., taking advantage of promotions, buying in bulk), commercial behaviours (e.g., flexing or cross-subsidising prices across products or brands), or the effects of co-occurring education, regulation of product labelling, advocacy campaigns, and media coverage may have influenced their observed outcomes [4,5].

Public health scientists have increasingly highlighted the importance of taking context into account in evaluating population interventions [17]. It has also been suggested that such interventions may be better considered as events in complex, adaptive systems, rather than within simpler linear causal models [18]. Systems thinking prompts us to ask different questions, which in turn leads to different kinds of evaluations. For example, in the case of the Chilean SSB tax, instead of asking ‘What are its impacts on price and sales?’ we would ask ‘What are the diverse impacts of announcing and introducing the tax, how do these come about, and how do they interact over time?’ While still considering impacts on price and sales, this would also prompt us to think more broadly about the wide-ranging potential intended and unanticipated consequences of such a tax at all levels of society, in all sectors, and for all stakeholders. It also prompts us to consider the potential for change in outcomes as a continuous process, rather than a single event, consequent upon the continuous adaptation that occurs within food systems in response to external stimuli. While such approaches to evaluation remain in their infancy, some valuable examples are emerging, such as a recent study evaluating the impacts of a voluntary scheme in which retailers were challenged to reduce availability of low cost, high strength beers and ciders [19,20]. In this study, the researchers engaged with the complexity of the context and the potential for wide-ranging consequences of the intervention, assessing these through multiple methods and diverse sampling strategies, as well as through analyses informed by complexity theory and systems thinking.

## Meeting the challenges

Broader, more nuanced, and more informative research questions cannot be answered using traditional, more simplistic approaches to evaluation. The present evidence base is significantly limited by a failure to embrace different ways of thinking and working, including addressing questions of context and those related to complex adaptive systems [17,18]. However, a consequence of taking a broader approach to evaluation that embraces complexity will be that it challenges prevailing methodological orthodoxies. Researchers, funders, and journals may all be reluctant to relinquish existing hierarchies of evidence and ‘traditional’ methods of evidence synthesis. With so many intractable global public health challenges associated with NCDs in need of robustly researched solutions, this seems short-sighted. Greater ambition and leadership are needed among researchers, funders, and policymakers to enable smarter approaches to the development and evaluation of low-agency population interventions, including taxes on unhealthy commodities, regulation of marketing, and structural interventions to make active living easier. Ambition and leadership coupled with greater international collaboration to identify opportunities for—and to coordinate efforts to fund, implement, and build capacity for—quasi-natural and natural experimental evaluations of these interventions could more rapidly advance science on NCD prevention.

## Abbreviations

NCD: non-communicable disease
SSB: sugar-sweetened beverage
